# Toward a new data standard for combined marine biological and environmental datasets - expanding OBIS beyond species occurrences

**DOI:** 10.3897/BDJ.5.e10989

**Published:** 2017-01-09

**Authors:** Daphnis De Pooter, Ward Appeltans, Nicolas Bailly, Sky Bristol, Klaas Deneudt, Menashè Eliezer, Ei Fujioka, Alessandra Giorgetti, Philip Goldstein, Mirtha Lewis, Marina Lipizer, Kevin Mackay, Maria Marin, Gwenaëlle Moncoiffé, Stamatina Nikolopoulou, Pieter Provoost, Shannon Rauch, Andres Roubicek, Carlos Torres, Anton van de Putte, Leen Vandepitte, Bart Vanhoorne, Matteo Vinci, Nina Wambiji, David Watts, Eduardo Klein Salas, Francisco Hernandez

**Affiliations:** 1 Vlaams Instituut voor de Zee / Flanders Marine Institute, EurOBIS, Oostende, Belgium; 2 UNESCO Intergovernmental Oceanographic Commission, Ocean Biogeographic Information System, UNESCO-IOC Project Office for IODE, Oostende, Belgium; 3 Hellenic Centre for Marine Research, MedOBIS, Gouves, Greece; 4 United States Geological Survey, OBIS-USA, Virginia, United States of America; 5 Istituto Nazionale di Oceanografia e di Geofisica Sperimentale (OGS), Trieste, Italy; 6 Marine Geospatial Ecology Laboratory, Nicholas School of the Environment, Duke University, OBIS-SEAMAP, Durham, NC, United States of America; 7 University of Colorado Museum of Natural History, OBIS-USA, Colorado, United States of America; 8 Centro Nacional Patagónico, ArOBIS, Puerto Madryn, Argentina; 9 National Institute of Water and Atmospheric Research, Southwest Pacific OBIS, Wellington, New Zealand; 10 British Oceanographic Data Centre, Liverpool, United Kingdom; 11 Woods Hole Oceanographic Institution, Woods Hole, United States of America; 12 CSIRO Oceans and Atmosphere, OBIS-Australia, Hobart, Australia; 13 Universidad Autónoma de Baja California, Mexicali, Mexico; 14 OD Nature, Royal Belgian Institute for Natural Sciences, Antarctic OBIS, Brussels, Belgium; 15 Kenya Marine and Fisheries Research Institute, Mombasa, Kenya; 16 Universidad Simon Bolivar, Caribbean OBIS, Caracas, Venezuela

**Keywords:** Darwin Core Archive, sample event, species occurrence, environmental data, ecosystem data, telemetry data, data standardisation, oceanographic data

## Introduction

The Ocean Biogeographic Information System (OBIS) was established in 2000 as a project of the Census of Marine Life (CoML) with the goal to create “an online, user-friendly system for absorbing, integrating, and accessing data about life in the oceans” (Grassle and Stocks 1999; Grassle 2000; Decker and O’Dor 2002; Yarincik and O'Dor 2005). At that time there was no global registry of marine species and no global repository for their occurrence data. OBIS provided the repository, and the World Register of Marine Species (WoRMS), which was launched in 2007 as a common source of species and their various names (Worms Editorial Board 2016), now provides the taxonomic backbone of OBIS.

After the successful decade-long CoML project, OBIS found a new hosting institution when in June 2009, the Member States of the Intergovernmental Oceanographic Commission (IOC) of UNESCO adopted OBIS as part of its International Oceanographic Data and Information Exchange (IODE) programme. Since then OBIS received recognition for its contribution to marine scientific research at the highest political level, through resolutions 69 and 70 of the United Nations General Assembly (Oceans and the Law of the Sea in the General Assembly of the United Nations 2015, Oceans and the Law of the Sea in the General Assembly of the United Nations 2014). OBIS also moved from a purely scientific endeavour to one that also supports monitoring, assessment and conservation in the marine environment.

The OBIS secretariat, hosted at the UNESCO/IOC project office for IODE in Oostende (Belgium), builds the central database (iOBIS) and fosters global benefits of the OBIS network through leadership in training, standards development, and international cooperation. OBIS now provides taxonomically and geographically resolved data for over 47 million observations of 116,000 marine species integrated from more than 2,000 databases, representing 600 institutions connected through 20 national, regional and thematic nodes. The data in OBIS continue to grow in the range of millions of records per year, and about 100 scientific publications cite OBIS annually.

This level of data integration requires strict application of internationally agreed standards. In 2005, OBIS agreed on a data standard by developing a specialized form of the internationally recognised Darwin Core (DwC) (Wieczorek et al. 2012), already in use at the time by the Global Biodiversity Information System (GBIF) and other biogeographic data communities.

In 2009, the Executive Committee of the Biodiversity Information Standards, also known as the Taxonomic Databases Working Group (TDWG), announced their ratification of an updated version of Darwin Core as a TDWG Standard. Ratified Darwin Core unifies specializations and innovations emerge from diverse communities, and provides guidelines for ongoing enhancement. The Darwin Core Quick Reference Guide links to TDWG’s term definitions and related practices for Ratified Darwin Core.

In December 2013, the 3rd session of the IODE Steering Group for OBIS agreed to transition OBIS globally to the TDWG-Ratified version of Darwin Core. This transition ensured that OBIS grows in a globally adopted context of up-to-date term definitions. It also enabled OBIS to use the Integrated Publishing Toolkit (IPT) (Robertson et al. 2014), developed by GBIF upon the definitions and practices embodied in Ratified Darwin Core, as the standard mechanism for the exchange of data and metadata between regional and thematic OBIS nodes and the central iOBIS node.

Data collected as part of marine biological research often include observations on habitat and physical and chemical features of the environment, as wel as details regarding the nature of the sampling or observation methods, equipment, and effort. Data sources often record these important types of data together, in what we refer to in this paper as "combined datasets".

OBIS participants often experience situations where biological data become disassociated from companion physico-chemical data and are sent to different repositories. Upon request from the OBIS secretariat and its European node (EurOBIS) hosted at the Flanders Marine Institute (VLIZ, Belgium), the IOC Committee on IODE (23rd session, March 2015, Brugge) recognised the need to investigate and develop these practices for combined datasets, and established a 2-year pilot project “Expanding OBIS with environmental data (OBIS-ENV-DATA)”. The project involved an international network of 11 institutions from 10 countries in North America, South America, Europe, Africa and Oceania.

Darwin Core-based communities have long recognised requirements of capturing expanded biological details, associated environmental details, and more information about methods. In 2013, GBIF hosted a "Hackathon" to bring together various communities to discuss their requirements and use cases and propose solutions (Wieczorek et al. 2014). In 2015, these developments culminated when GBIF released IPT technology that enables a new "core type": the Event Core. The Event Core goes beyond occurrence-based records, such as those OBIS currently serves (“Occurrence Core”), by combining a new Darwin Core “core type” with extensions to make the sampling event the central data entity linking biological, environmental, and sampling information.

This paper is the result of the OBIS-ENV-DATA workshop held in Oostende on 5-7 October 2015, and subsequent discussion, design, issue resolution, and technical development. This paper describes the OBIS-ENV-DATA pilot project evaluation and decision among specific alternatives to effectively manage combined datasets for scientific applications.

## Pilot Datasets

The OBIS-ENV-DATA consortium members were invited to submit pilot datasets. Datasets taken into consideration contained both abiotic and biotic data that had been analyzed together (“combined datasets”), datasets derived from automated readings (e.g., telemetry data), and datasets containing biometric measurements of specimens. Priority was given to those datasets that had been used in scientific publications.

The project partners submitted 13 pilot datasets (Table 1), which cover a biological diversity of 6 functional groups and include abundance, biomass and biometrical readings, as well as abiotic variables ranging from sediment characteristics (e.g., percentage of clay, concentration of copper, …), to water sample characteristics (e.g., concentration of NO_3_, concentration of chlorophyll a, salinity, temperature, …), environmental parameters (e.g., wind direction, ice coverage, light conditions, solar azimuth, secchi disk depth, bottom temperature, surface salinity) and sensor derived parameters (e.g., data from conductivity-temperature-depth (CTD) devices, tracking sensor, sensors attached to a sampling net, …).

Based on the available abiotic data, datasets were grouped into four categories:

1. **Sample based**: Both abiotic and biotic data are derived from measurements in samples, for example chlorophyll a concentration in a Niskin Bottle.
2. **CTD and CTD-like**: CTD sampling provides many readings of the same parameter along a depth profile. Datasets belong to the CTD-like group when a CTD profile was measured at the same date/time and location as a biological sample was taken, or when a sensor was placed on the gear used to collect the biological sample.
3. **Tracking and Telemetry**: Tracking data is obtained by placing a device on an animal (typically birds, turtles, large fish and mammals) that enables following that individual’s course in space and time. Telemetry datasets contain temporal and geographical animal tracking information plus abiotic parameters (like temperature and salinity) obtained by the tracking device. These datasets may also include biometric information, measured during placement of the sensor.
4. **Video Plankton Recorder** (**VPR)**: A Video Plankton Recorder is a device towed by a research vessel and equipped with a high resolution underwater digital camera system that takes images of passing water at high frequency (typically 30 images per second).

## The Darwin Core Archive

Central to OBIS's use of Darwin Core, for standard content and for effective data flow, is the role of the GBIF Integrated Publishing Toolkit (IPT) and the Darwin Core Archive (DwC-A) format for packaging components of Darwin Core biodiversity information in a single, self-contained dataset (GBIF 2010). The IPT software package helps its users to format their data into a DwC-A file.

The conceptual data model of the Darwin Core Archive is a “star schema” (Robertson et al. 2014), with a core record, such as an occurrence or an event, as the center of the star. Extension records, radiating out of the star, can optionally be associated with the core, linked by database keys such as an ID column. Column names in the records map to Darwin Core terms. In the star schema, there is only one center of the star, so the entire schema is only two levels deep: a single core with zero, one, or many extensions. Each core-to-extension relationship can be one-to-one, where there is only one extension record for each core record - also called “Simple Darwin Core” (Wieczorek et al. 2015) - or one-to-many, where for example many environmental observation records, or many biological occurrence records, can be associated with a single sampling event.

The IPT software assists the user in mapping data to valid Darwin Core terms and archiving and compressing the Darwin Core content with: (i) a descriptor file (meta.xml) that describes the mappings, and (ii) a dataset level metadata document in Ecological Metadata Language (eml.xml). These components, compressed together, comprise the Darwin Core Archive (Robertson et al. 2014).

Particularly of interest for the OBIS-ENV-DATA pilot project are the DwC **Occurrence** Core, the DwC **Event** Core, and the DwC **MeasurementOrFact** (MoF) Extension. A comprehensive description of the available column headers is provided by GBIF at http://rs.gbif.org/.

### Occurrence Core and Event Core

The DwC Occurrence Core contains terms to capture information about the existence of an organism at a particular place at a particular time. It can be extended with a MoF Extension in which specific measurements or facts related to the occurrence can be recorded.

The DwC Event Core contains terms to describe multiple types and multiple records of information associated with location and time. The Event Core can be used to record data resulting from a sampling event combined with the Occurrence Extension (which is identical in structure to the Occurrence Core) to associate information about organisms found in the sample. This format allows for more efficient storage of the sample data, as one event can be linked to several occurrences. In addition, the Event Core combined with the MoF Extension can integrate measurements or facts relating to the event. However, due to the star schema inherent to the DwC-A format, it is not possible to use the MoF Extension to store measurements or facts related to the occurrence when using the Event Core (GBIF/EU BON 2015).

One solution to this limitation of the star schema would be to store all occurrence related information in a structured format (e.g., JSON) in the column dynamicProperties of the Occurrence Extension. We did not pursue this option for several reasons: (i) it would be challenging for many people to produce this format; (ii) it would be difficult for less experienced data users to access these data; (iii) it would complicate the exercise of parameter standardization we discuss later; and (iv) OBIS would need to be able to automatically extract this structured data in order to analyze it.

### Event hierarchy

The inclusion of the column parentEventID in the Event Core makes it possible to create hierarchies among events. GBIF provides an example that describes how the parentEventID can be used to link smaller plots (subplots) to a larger plot (parent plot). This practice makes it possible to accurately describe, for example, whether a specimen was recorded during the analysis of an entire Whittaker Plot or during the analysis of one of the different subplots. This type of hierarchy makes it unnecessary to repeat information at the child event level (GBIF/EU BON 2015; Global Biodiversity Information Facility 2016).

The format for combined datasets proposed here embraces this development. In biological oceanography, for example, data are often gathered using research vessels that visit several stations during a given cruise and deploy different instruments at each station. The event hierarchy allows for the creation of one event record for each cruise (parent event), one event record for each visit to a station (child event), different event records for each sampling activity at a station (grandchild events) and, if applicable, different event records for subsamples (great-grandchild events).

**Figure 1.**
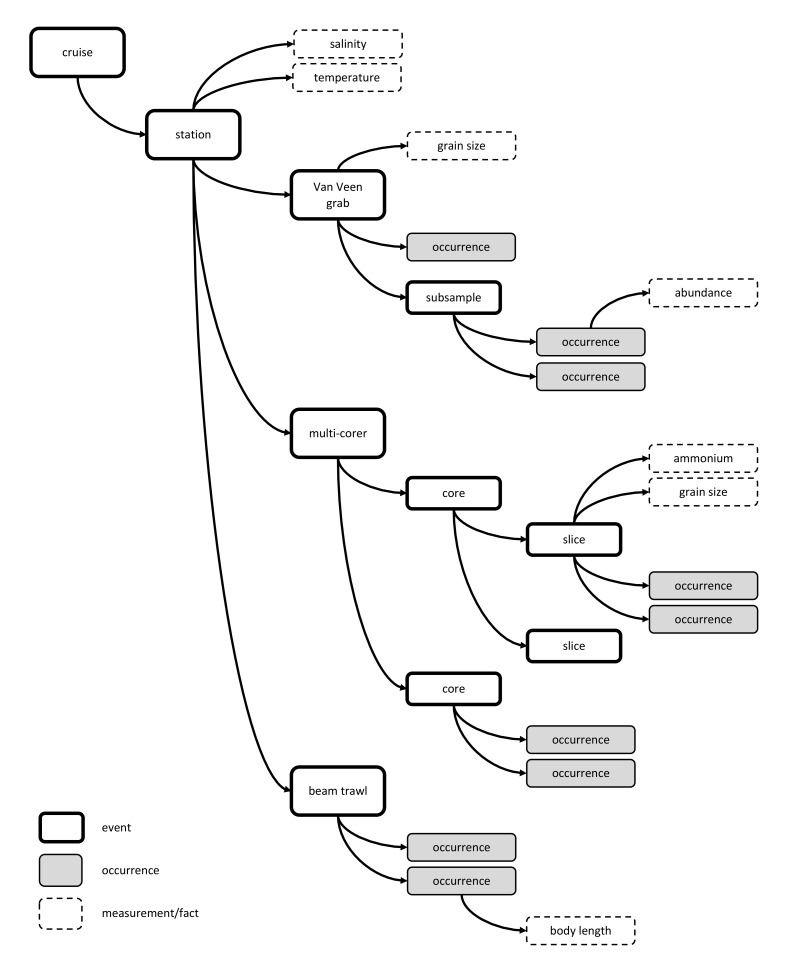
A hypothetical example based on a complicated sequence of sampling events at a given sampling location. In the example the bold rectangles are sampling events, the dashed rectangles measurements or facts, the grey rectangles are occurrences. The arrows between the rectangles illustrate the (hierarchical) relations between the different sampling events and between events and their associated occurrences and measurements. The example shows data sampled using a Van Veen grab, a beam trawl, and a multi-corer. The macrobenthos analysis was based on the complete Van Veen grab sample, while the meiofauna analysis was based on subsamples. The multicore sample was divided into different depth slices. Likewise, an abiotic measurement can refer to the entire sample or to a subsample.

As will be discussed later, different samples taken at the same sampling site may differ in coordinates although they are meant to be analysed together. In oceanography the depth at which a sample was taken or an environmental reading occurred is very relevant. The event hierarchy makes it possible to record differences in sampling time, location and depth while grouping these samples together in the same station visit. In addition, there is the added benefit of keeping all data at the appropriate levels and thus reducing data duplication to a minimum. An example of a hypothetical dataset with a complicated sequence of sampling events is provided in Fig. 1.

## Formatting combined data into a Darwin Core Archive: 6 proposed options and their feasibility

There can be more than one way to design a Darwin Core solution to challenges of combined data. Prior to the workshop, four options were identified and the pilot datasets were transformed to one or several of these formats to illustrate and explore potential usages and issues. During the workshop two more alternatives were identified and all six choices were discussed and evaluated.

### Option 1: Occurrence Core combined with MoF Extension

**Figure 2.**
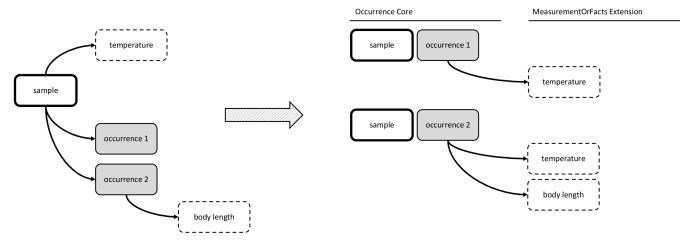
A schematic diagram of Option 1: Occurrence Core combined with MoF Extension. The diagram shows that the temperature measurement is duplicated in the MoF Extension for each occurrence record of the same sample.

**Figure 3.**
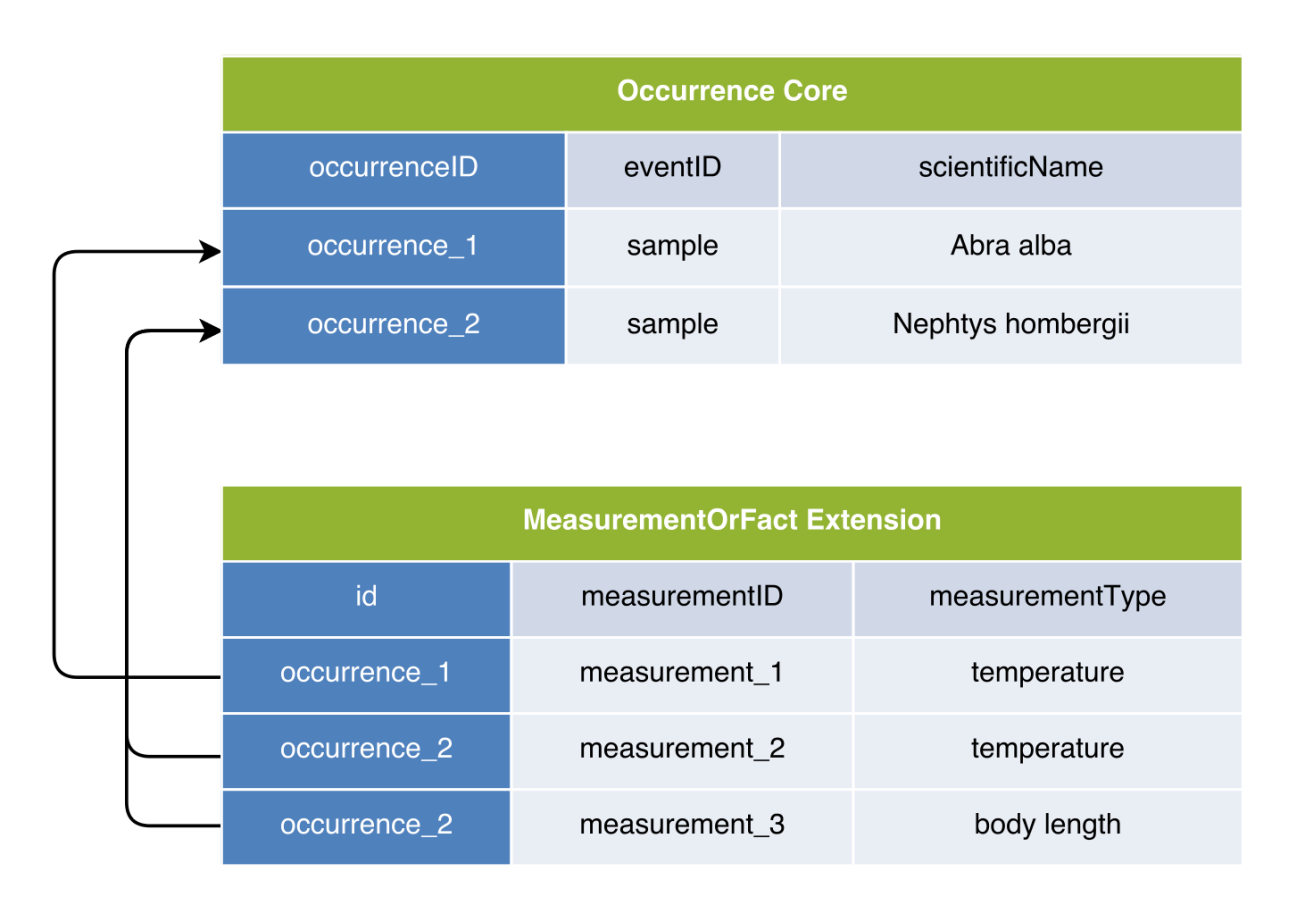
A practical example of Option 1: Occurrence Core combined with MoF Extension. The temperature measurement is duplicated for both occurrences.

In the first option, the DwC Occurrence Core is used in combination with the MeasurementOrFact (MoF) Extension. In this option all parameters which do not fit in the Occurrence Core can be captured as different measurementTypes, linked in a many to one relation with the Occurrence Core record. In this manner, the MoF Extension may include both abiotic and biotic parameters linked with the organism observed in the occurrence record as is illustrated in Figs 2, 3.

Option 1 was only a good solution for datasets that did not include associated abiotic data (e.g., the “*Sizing Ocean Giants*” dataset), because abiotic measurements need to be duplicated for each occurrence record belonging to the same sample. This duplication in itself may only be an inconvenience, because the DwC-A is designed for data exchange, and systems harvesting the data may store them in a more efficient format. However, more serious problems with Option 1 became apparent when abiotic and species occurrence data originated from different samples taken at different times and locations, as was demonstrated for the dataset “*Hyperbenthic communities of the North Sea.*” In this dataset, a Sorbe sledge was towed to sample the hyperbenthos and a multi-corer was deployed to sample the substrate. As a sledge is towed over a long distance (typically a few kilometres), and the sediment properties may differ over this transect, the exact coordinates of deployment of the multi-corer are very relevant. As abiotic measurements are linked directly to occurrence records, temporal and geographic differences between biotic and abiotic samples cannot be captured using Option 1. A solution for this problem could be to introduce *event occurrence records* and an event hierarchy into the occurrence core, which is discussed in this paper as option 4.

### Option 2: Event Core combined with Occurrence Extension and MoF Extension

**Figure 4.**
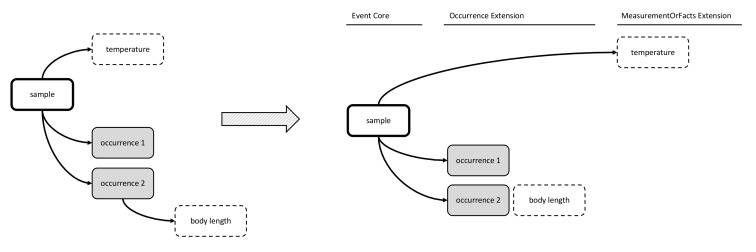
A schematic diagram of Option 2: Event Core combined with Occurrence Extension and MoF Extension. The diagram shows that the body length measurement needs to be integrated in the occurrence extension.

**Figure 5.**
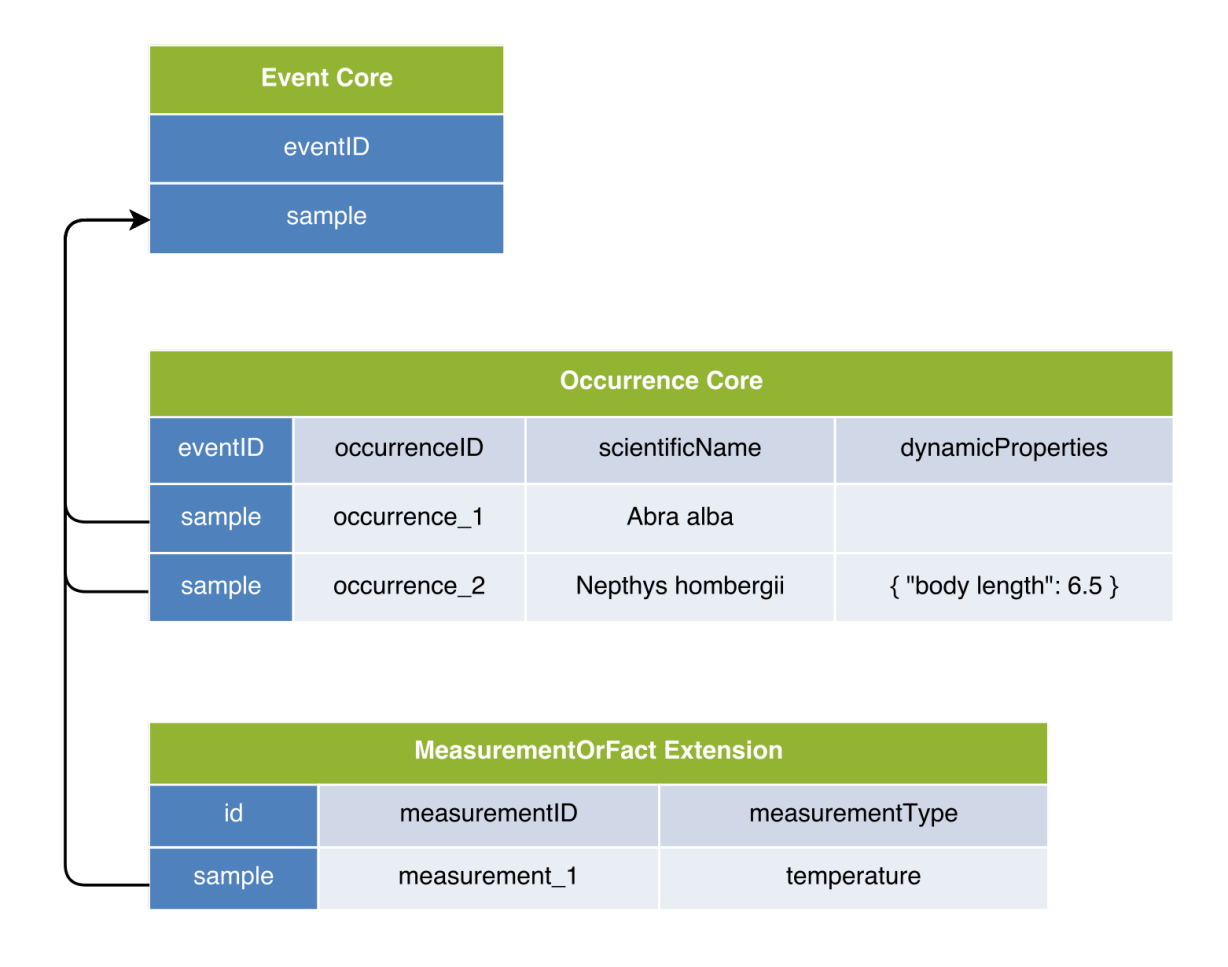
A practical example of Option 2: Event Core combined with Occurrence Extension and MoF Extension. As the body length measurement can't be stored in the MoF Extension, it is stored in the column dynamicProperties of the Occurrence Core instead.

The second option made use of the Event Core, which was released by GBIF in September 2015 as a new feature of DwC-A in order to efficiently capture sample data (GBIF/EU BON 2015). The Event Core includes all the DwC event-based data columns from the DwC Occurrence Core, and is to be used in combination with a DwC Occurrence Extension and a MoF Extension. The occurrences and measurement types are linked in a one-to-many relationship with Event Core records. In this option, the MoF Extension only links to the event, hence it can only be used for parameters relating to the event (meaning abiotic data) and not for biological parameters, which need to be integrated in the occurrence record, as is shown in Figs 4, 5.

Option 2 avoids the data duplication associated with Option 1, as abiotic measurements can be linked directly to the event and do not need to be repeated for every occurrence record. This option also allows the creation of a separate event record for an abiotic sample and to record different coordinates or times for the biotic and abiotic samples. However, this option removes the possibility of including biometric measurements and necessitates the use of the DwC field quantification type to record abundances or biomasses in the Event Core instead of in the MoF Extension. This is not ideal for three reasons: (i) if a dataset includes both abundance and biomass of a single sample, two different occurrence records will have to be created for the same species occurrence; (ii) there may be additional biological details desirable to capture in MoF, beyond just organism quantity; and (iii) the MoF Extension has some additional valuable terms, that are missing from the Occurrence Core. For example, if a dataset contains abundances recalculated to a standard unit, this information cannot be added elegantly in the Occurrence Extension. This could be dealt with in a variety of ways, none of which are ideal, including: adding the units of quantification in the DwC field organismQuantityType (e.g., abundance per m²); providing the units of the parameter instead of the actual sample size in the DwC field sampleSize; or attempting to recalculate the original abundance value from the standardised parameter and use this value in combination with the sample size (e.g., individuals per 0.5 m^2^).

### Option 3: Event Core combined with MoF Extension & Occurrence Core combined with MoF Extension

**Figure 6.**
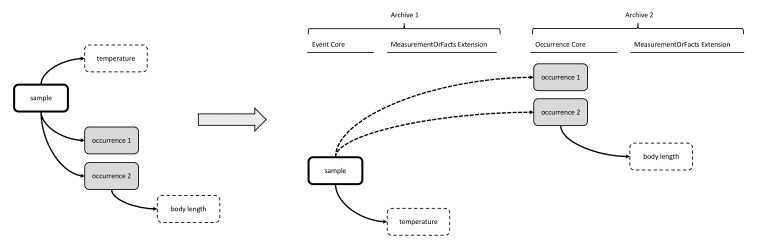
A schematic diagram of Option 3: Event Core combined with MoF Extension & Occurrence Core combined with MoF Extension. The diagram shows that this approach combines the best features of Option 1 and Option 2, but has the downside of separating the abiotic from the biotic data into different resources.

**Figure 7.**
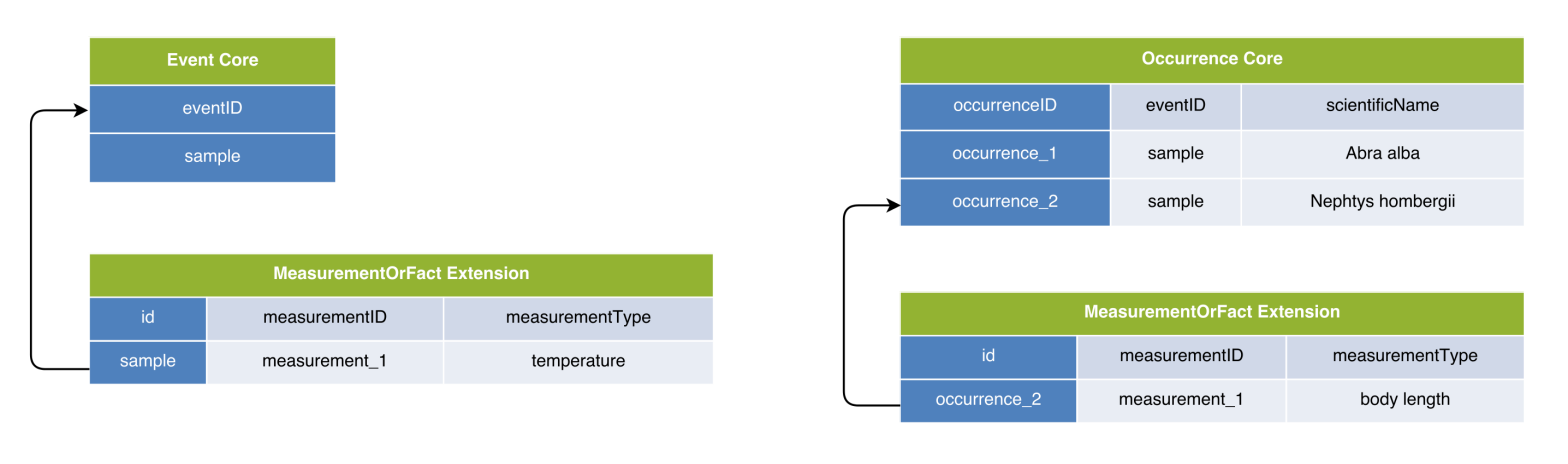
A practical example of Option 3: Event Core combined with MoF Extension & Occurrence Core combined with MoF Extension. This approach combines the best features of Option 1 and Option 2, but has the downside of separating the abiotic from the biotic data into different resources.

Option 3 separates the biological data and the environmental data into different IPT resources or Darwin Core Archives, which can be linked by assigning them the same “Collection Name” and/or “Collection Identifier” in the EML metadata. The biological data would be stored using the Occurrence Core (Option 1), and the abiotic data using the Event Core (Option 2), making Option 3 essentially an approach to combine benefits of Options 1 and 2. Both resources would need to use the same eventIDs for the same events to link individual records of abiotic data correctly with their corresponding biotic data, as is illustrated in Figs 6, 7.

Option 3 was appealing during the workshop evaluation, because it uses the best features of both Event Core and Occurrence Core, and thus solves the problems associated with Option 1 and Option 2. In biological oceanography, data from the same physical sample are sometimes separated into different subsamples so the different functional groups can be analysed by different research groups. An additional benefit of Option 3 is that it allows such subsets to remain separate Occurrence Core-based DwC-A resources, each referring to the same sampling event (stored in the Event Core-based DwC-A resource).

There are however some problems with option 3: (i) due to technical constraints at the time of the workshop, it was not possible to use Event Core solely for abiotic data because the IPT required the use of an Occurrence Extension in combination with Event Core. However, this constraint has since been removed; (ii) some concern was expressed that separating the data into different resources may be prone to error, because in order to ascertain that the different resources link to each other and use the same eventIDs for the same events, all resources would need to be created or reviewed at the same time; (iii) it may also be unclear to users that abiotic data exist when they are in different IPT resources, although this can be solved by clearly documenting this in the IPT EML metadata, and (iv) another concern was that for automated harvesters such as OBIS, it may prove challenging to automatically account for this separation and linkage in their harvesting procedures.

### Option 4: Occurrence Core combined with MoF Extension; each event is documented as a separate record in the Occurrence Core

**Figure 8.**
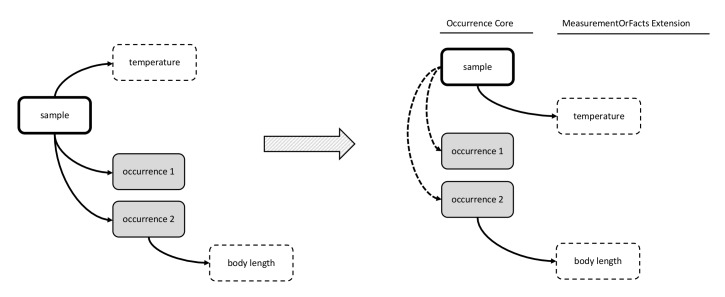
A schematic diagram of Option 4: Occurrence Core combined with MoF Extension; each sampling event is documented as a separate record in the Occurrence Core. The diagram shows that this format allows efficient storage of both biotic and abiotic measurements in the same DwC-A, but it increases complexity by introducing an event record without associated occurrence information into the Occurrence Core.

**Figure 9.**
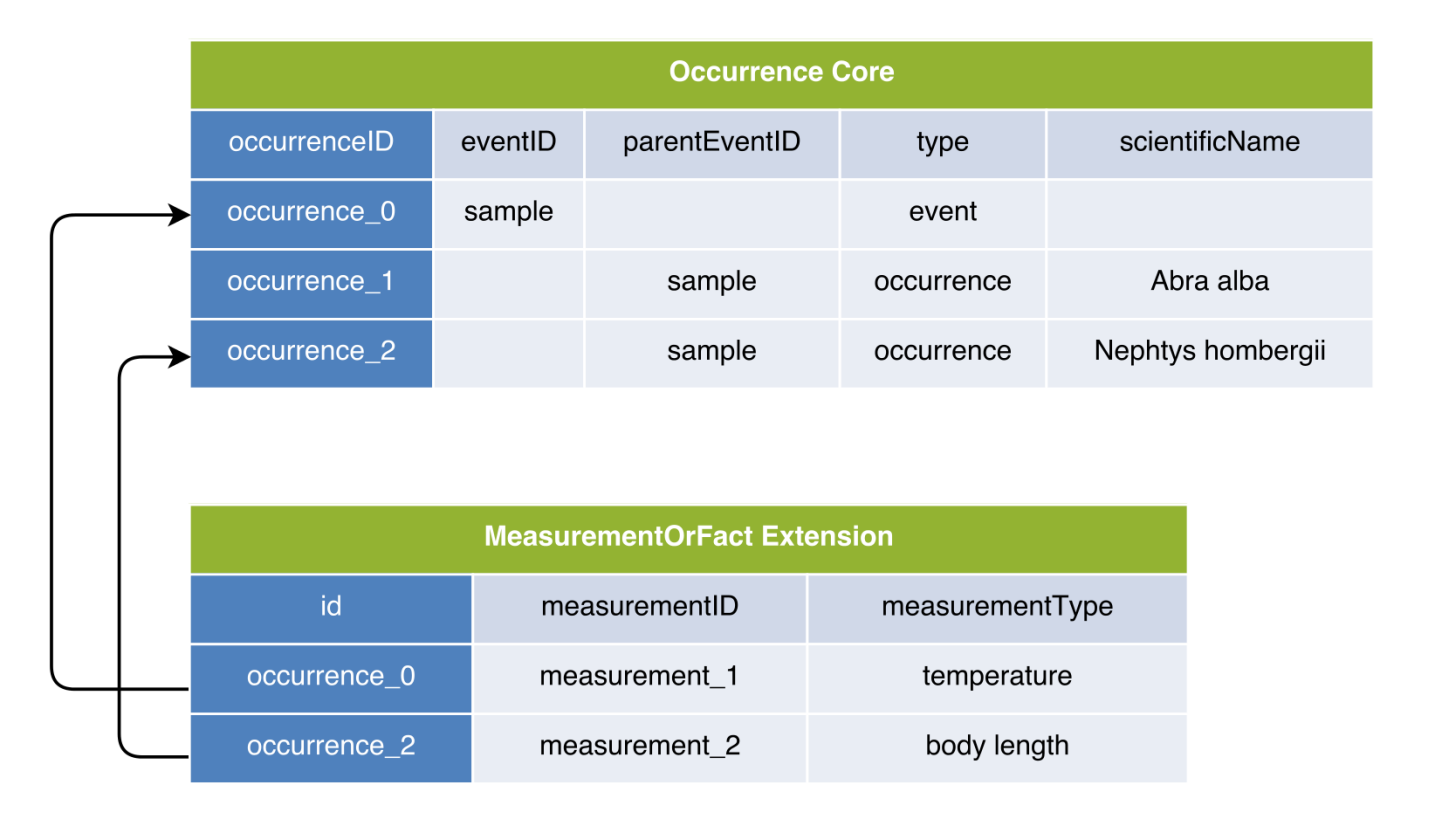
A practical example of Option 4: Occurrence Core combined with MoF Extension; each sampling event is documented as a separate record in the Occurrence Core. This example demonstrates that this format allows efficient storage of both biotic and abiotic measurements in the same DwC-A, but it increases complexity by introducing an event record without associated occurrence information into the Occurrence Core.

The fourth option uses the Occurrence Core in combination with the MoF Extension. It differs from Option 1 as for each event an extra occurrence record is created which links to the event-based abiotic data in the MoF Extension. This *event occurrence record* doesn't contain any species occurrence information but is linked to actual occurrences by using the columns eventID and parentEventID. Additionally, one can specify "event" in the DwC field type to distinguish this record from the species occurrence records as is illustrated in Figs 8, 9.

Data can be stored in this format without any of the problems associated with Options 1, 2 or 3. However, Option 4 was controversial as it forces the Occurrence Core to include event records without an associated species occurrence. Many workshop participants felt that this practice may be counter-intuitive to both users and data managers and therefore prone to errors. As with Option 3, this might also pose challenges for automated data harvesters.

### Option 5: Event Core combined with Occurrence Extension and MoF Extension; each occurrence is documented as a “dummy” event in the Event Core

**Figure 10.**
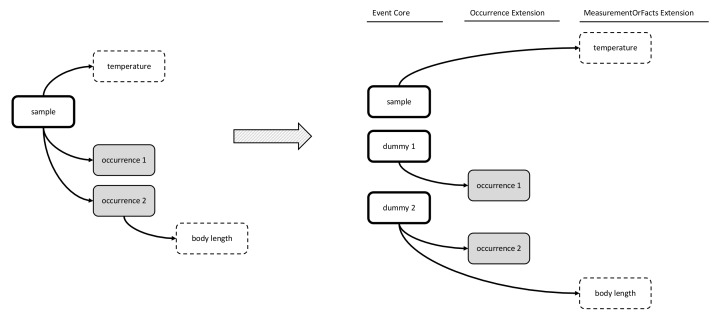
A schematic diagram of Option 5: Event Core combined with Occurrence Extension and MoF Extension; each occurrence is documented as a “dummy” event in the Event Core. The diagram shows that this format allows storage of both biotic and abiotic measurements in the same DwC-A, but it greatly increases complexity by introducing dummy records into the Event Core.

**Figure 11.**
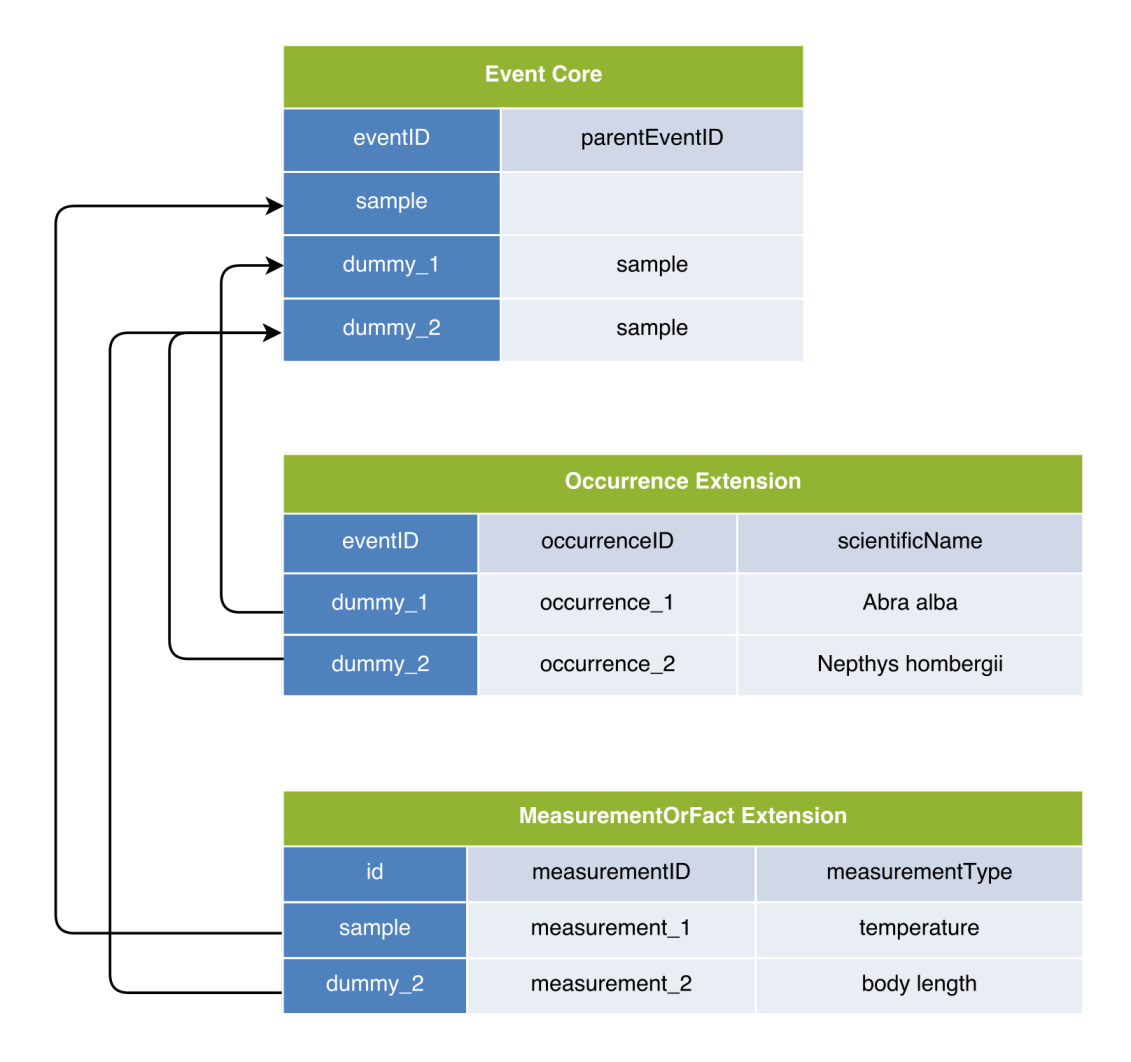
A practical example of Option 5: Event Core combined with Occurrence Extension and MoF Extension; each occurrence is documented as a “dummy” event in the Event Core. This format allows storage of both biotic and abiotic measurements in the same DwC-A, but it greatly increases complexity by introducing dummy records into the Event Core.

Option 5 emerged during the workshop and may be considered the inverse of Option 4. It uses Event Core, the Occurrence Extension and the MoF Extension, but it differs from Option 2 in that it includes additional ‘dummy events’ in the Event Core, as if each occurrence was a separate event. The dummy events link to the Occurrence Extension in a one-to-one relation, and may also link to the MoF Extension. Option 5 uses the parentEventID to link the dummy events to the samples in which the occurrences were encountered. This workaround allows the MoF to capture both abiotic (linked to the eventID of the sample) and biotic measurements (linked to the dummy eventID) as is demonstrated in Figs 10, 11.

Option 5 allows for both biotic and abiotic information to be stored in the MoF Extension with perhaps less abuse of the system compared to Option 4. However, creating a dummy event for each occurrence results in a complicated and unintuitive hierarchical structure of events in the Event Core.

### Option 6: Event Core combined with Occurrence Extension and a newly proposed MoF Extension; both Extensions are linked, based on an OccurrenceID

Discussing and testing the previously identified options repeatedly led the workshop participants to see the DwC-A star schema limitation as a central challenge to meeting requirements with Event Core. The team perceived that a simple link between the occurrence and MoF Extensions would greatly simplify the format and this ultimately led to Option 6.

**Figure 12.**
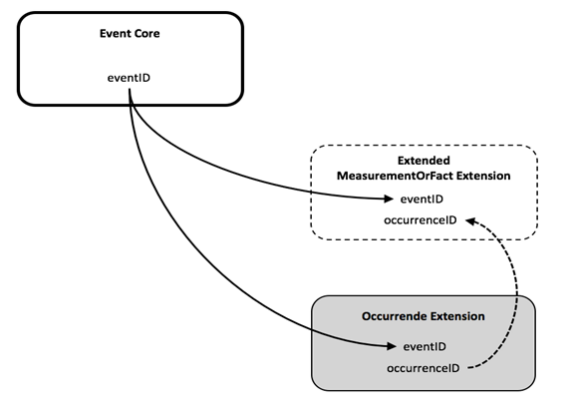
Schematic diagram showing linkages between the Event Core, the Occurrence Extension and the ExtendedMeasurementOrFact Extension. The IPT's enforcement of the star schema is not broken, but the DwC-A capability is extended with an additional link between both extensions for biotic measurements.

**Figure 13.**
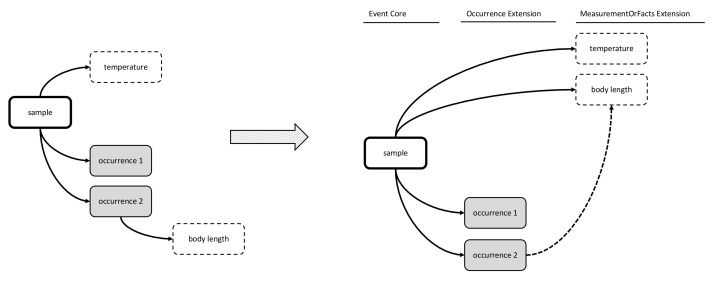
A schematic diagram of Option 6: Event Core combined with Occurrence Extension and a newly proposed MoF Extension; both extensions are linked with an OccurrenceID. The diagram shows that this is the most simple option to efficiently store both biotic and abiotic measurements in the same DwC-A.

**Figure 14.**
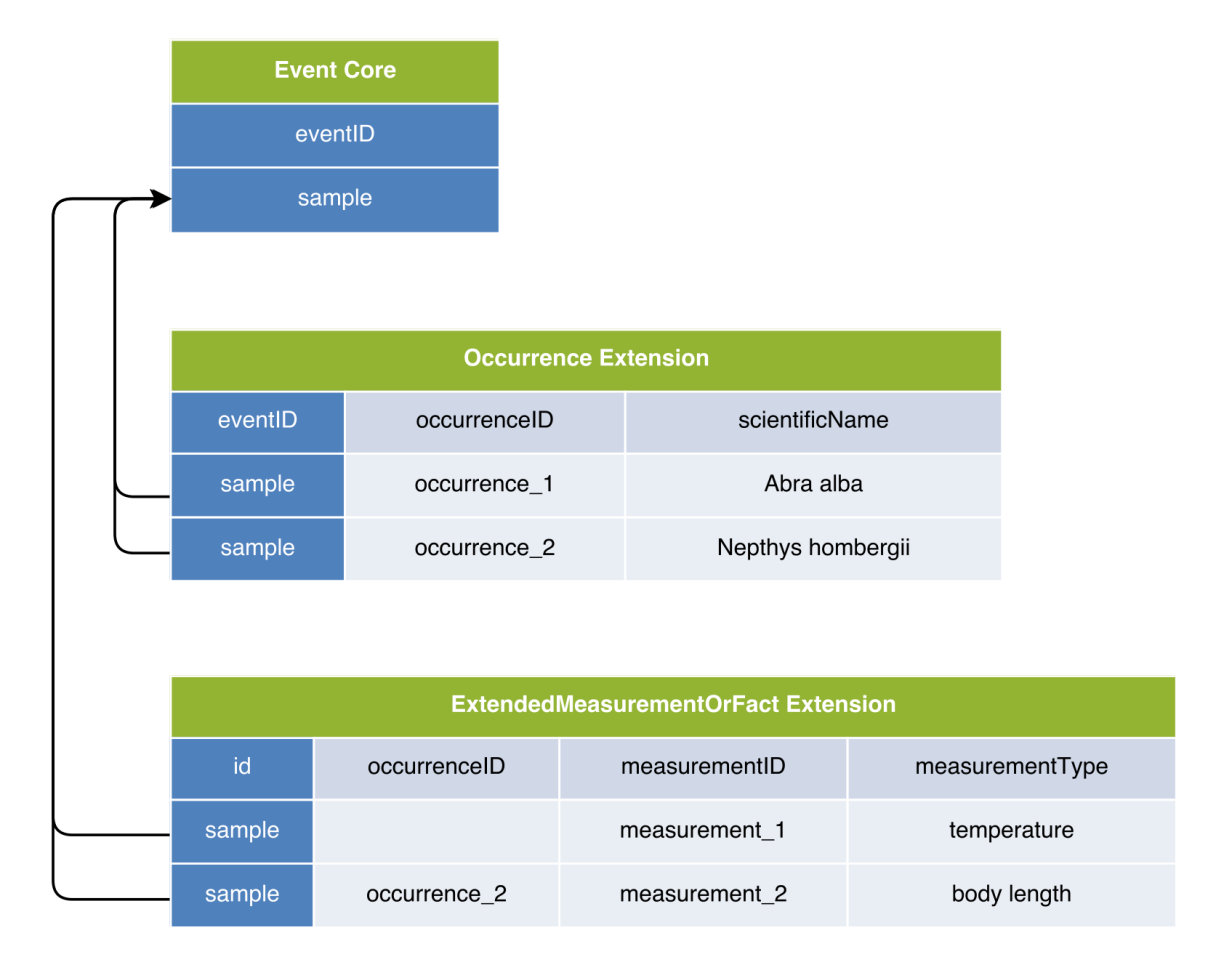
A practical example of Option 6: Event Core combined with Occurrence Extension and a newly proposed eMoF Extension; both extensions are linked with an OccurrenceID. This format is the most simple option to efficiently store both biotic and abiotic measurements in the same DwC-A.

Option 6 includes the Event Core and the Occurrence Extension. It differs from the other options in that it introduces a customized MoF Extension (hereafter referred to as the extended or “eMoF” Extension) which was extended with an additional column, occurrenceID. Biotic measurements in the eMoF will be assigned both an eventID and an occurrenceID, while abiotic measurements will only receive an eventID as is illustrated by Figs 12, 13, 14.

Initially Option 6 seemed to conflict with the DwC star schema and therefore to be incompatible with the IPT. However Option 6, as it was worked out during the workshop, did not break IPT's enforcement of the star schema and expanded the DwC-A capability with an additional link between the eMoF Extension and the Occurrence Extension.

### Selection of option 6 for combined datasets

The options discussed in this manuscript began to take shape during the preparation for the workshop, although the final form of all six was not complete until the workshop itself. Along the way, the pilot dataset activity provided examples, test cases, and creativity to refine the six options. Some sense of evaluation criteria - how can we select among the options? - grew before and during the workshop as well. The methods to compare the options became clearer by examples. During the workshop, the group discussed options intensely, refining each option to a consistent definition. The group thought through and discussed cases and implications hypothetically, and tested many using actual data from the pilot datasets.

Table 2 summarizes the method of evaluating the options. We considered advantages and disadvantages, as shown, for an open-ended approach to choosing among the options. We also developed two more fixed criteria, summarized as (1) ease of training and implementation, and (2) suitability of the resulting data structure to meet science and data management applications that are important to OBIS. Table 2 summarizes the opinions and ultimately the consensus of the group with respect to general assessment of advantages and disadvantages, and the consideration of the two specific criteria. Ultimately, we chose Option 6 because it is simple yet capable and flexible. It avoids the conceptual or operational complexity of some other options, and does not sacrifice or compromise features OBIS desires, such as event hierarchy, measurement format, and use of IPT.

The flexibility and ease of use of Option 6 for different types of combined datasets is demonstrated through a number of showcases. Pilot datasets from the four identified types (see above) have been formatted following the Option 6 structure, and are available at http://ipt.iobis.org/obis-env/.

## Event Hierarchy in Option 6

We embrace event hierarchy as it provides a solution for several of the problems discussed in the previous sections. Option 6 preserves the function of the Darwin Core event hierarchy, and enables OBIS to employ the event hierarchy effectively for important needs of the OBIS community.

For example, during a research cruise different stations may be sampled, different samples may be obtained at the same station, and some samples may be analysed in full to quantify some species groups, while for other species subsampling may take place. Consider the following scenario: a single station may be sampled using a Van Veen grab, a beam trawl, and a multi-corer. Samples from these three events are meant to be analysed together but may differ in geographic coordinates, sample depths and sample time, and therefore each need to be assigned a different eventID. Event hierarchy will allow these three *sibling* events to be linked to the same parentEventID for the station. While the grab and multi-corer can be represented by a lat/long point, creating a different eventID for the beam trawl will allow the track to be recorded as a line feature in the footprintWKT field. For core samples generated by the multi-corer, different depth slices of cores may be analysed separately to record the occurrence of biological specimens, while abiotic measurements may be recorded for all slices combined. The event hierarchy approach allows for the occurrence data within depth slices of cores to become child events of the core event, and grandchild events of the station. The occurrences and abiotic readings which were recorded for the entire sample can be linked directly to the (child) event for the entire sample. This elaborate example is presented as a schematic diagram in Fig. 1.

There might be an issue for situations, such as described above, where essential metadata such as coordinates and dates are not repeated at each sub-event. In those cases the human readability of the file is highly reduced. If no information is included to indicate the hierarchical level of each event, interpretation of the event file will become problematic. Hence, OBIS could recommend filling out the DwC column type to specify the level for each event. Proposed values include “cruise”, “station”, “sample”, and “subsample”. An additional way to ensure the readability of the event hierarchy could be to build a hierarchical structure into the eventID itself. For example, one could name an eventID referring to a subsample “station5:sample1:subsample2”, with parenteventID “station5:sample1,” with parenteventID “station5”. This approach has the benefit that if the data is sorted by eventID it will also be sorted according to the hierarchical structure, which will make it easier to understand by a human reader. An additional benefit is that simply by reading the eventID one will know the level of the event record in the hierarchy, making it easier for a human reader to assess the event record separately.

## Option 6 and sensor based data

A fairly recent development in marine sciences is that of biological data obtained by sensors. The readings by automated sensors can create huge amounts of data; for example, a sensor placed on a bird may provide a positional record every few minutes (Stienen et al. 2016); a video plankton recorder towed behind a research vessel may generate over a million presence/absence records in a single day (Ashjian et al. 2001). As these new types of data are becoming increasingly available, the importance of efficient data storage increases.

It was recognised during the workshop that the raw sensor data may not always be the data actually used during analyses. For example in the dataset “Gulf of Maine Zooplankton: Abundance and Biomass from MOCNESS tows,” sampling occurred at a very small depth interval, limiting the variation in the measured parameters. It would therefore be sufficient to only store the average values for temperature and salinity for each tow instead of the raw sensor data.

In the case of CTD data, scientists may sometimes only be interested in derived data, such as the thermocline depth, thermocline thickness, surface temperature, bottom temperature, etc. In such cases it may be preferable for the combined dataset to contain only these derived data in combination with the biotic data, rather than the entire CTD profile. These are data exchange protocol conventions that ideally should be agreed upon by the various observing communities. In the Option 6 format, the raw data as well as the derived data can be stored without problems.

In the following examples we explain how Option 6 can work for 3 different types of sensor data: CTD, Tracking, and Video Plankton Recorder data.

### CTD Data

For CTD data, the use of the Occurrence Core is particularly problematic, not only because a CTD cast typically has hundreds of readings, each of which would need to be duplicated for each occurrence, but also because the depth of each reading cannot be stored in an easily accessible manner. Often a CTD is attached to a rosette which holds Niskin bottles that are used to collect water samples at different depths. These samples can be analysed for example to estimate abundance of microplankton.

**Figure 15.**
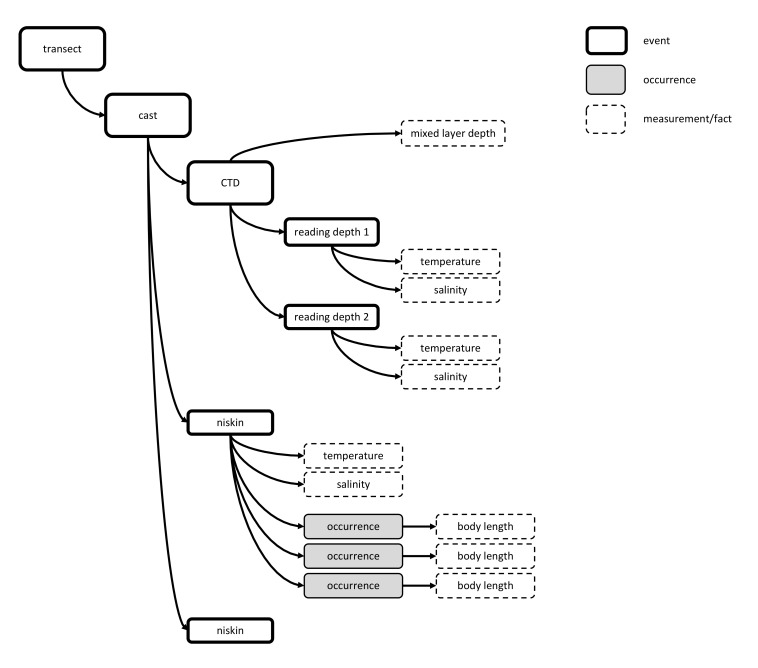
A schematic presentation of a sampling event using a CTD and several Niskin bottles mounted on a rosette. In the example the bold rectangles are sampling events, the dashed rectangles measurements or facts, the grey rectangles are occurrences. All CTD readings taken at the same time at the same depth are grouped together into a child event of the event "CTD". Data derived from the CTD measurements like "mixed layer depth" can be linked directly to the event "reading depth". Each sample taken by a Niskin bottle placed on the roset can be considered a different child event "niskin" of the event "cast". Measurements of the water collected by the Niskin bottle can be linked to these "niskin" events.

Option 6 - using the event hierarchy - avoids duplication, as all sensor readings occurring at the same time (and depth) can be considered a single child event of the event “CTD cast”. The water samples collected by Niskin bottles can also be included in the hierarchy as different child events as is illustrated by Fig. 15.

CTD sensors can be deployed in combination with other sampling devices. It may for example be attached to a plankton sampling net. In this case the parent event may represent the deployment of the net, while the child events would represent the sensor readings. This means that the biological data can be linked directly to the parent event.

### Telemetry data

One can simply use an Occurrence Core with MoF Extension (Option 1) to store raw tracking data and any associated abiotic and biotic data. However this requires that for each sensor reading all occurrence-based information be duplicated (scientific name, biometrics), while typically only the coordinates and possibly other sensor readings differ. It was also argued during the workshop that - from an OBIS perspective - it would be sensible to allow for some grouping of the occurrence records belonging to the same “phase”: for example, if an animal spends a certain period in one area this could be recorded within a single occurrence record.

Option 6, using the event hierarchy, provides an answer to both concerns. Each separate sensor reading can be considered a child event of a parent event which can be linked to an occurrence record for an animal. The parent event may represent the entire tracking sequence of an animal, or a grouping of sorts for readings recorded at the same period and area, which are then grouped to make up the entire track.

**Figure 16.**
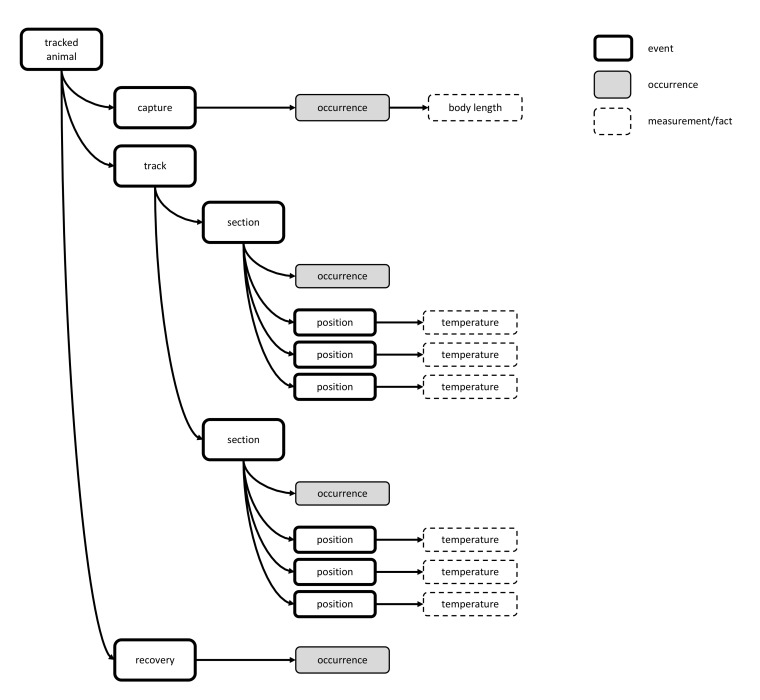
A schematic presentation showing how telemetry data can be stored using Option 6. In the example the bold rectangles are sampling events, the dashed rectangles measurements or facts, the grey rectangles are occurrences. A tracked animal can have 3 main child events: "capture", "tracking" and "recovery". Biometrical data are usually measured during or shortly after the capture, and can be linked to the occurrence of the "capture" event. A separate GPS sensor reading is considered as a child event "position" of the event "section". The "section" event represents a biologically meaningful grouping of sorts for readings recorded at the same period and area, and is linked to an occurrence record. Any abiotic data can be stored efficiently by linking the "position" event records with the eMoF table or to whichever level they are applicable. The "position" events are not linked with an occurrence record.

Any abiotic data can be stored efficiently by linking the child event records with the eMoF Extension. This method also allows the eMoF Extension to efficiently store the biometric data which is usually measured during tagging. An schematic diagram of how telemetry data can be stored is provided in Fig. 16.

This same format could also be used for data derived from tags placed on animals (typically fish). The difference between a tagging and a tracking dataset is that a tag does not send out a GPS signal, instead a stationary sensor detects the tag when it moves within range. Software can automatically combine all readings taken by a single sensor within a certain period (e.g., 2 hours). As with tracking datasets, the raw sensor readings could be grouped by a parent event record, which is used to link to the Occurrence Extension.

### Video Plankton Recorder (VPR)

A VPR is a device with a high resolution underwater digital camera system which is towed behind a vessel, recording images at a high frequency (typically 30 frames per second) of the water passing through it. These images are analysed and grouped by software which rapidly quantifies taxonomic composition.

In cases where the VPR was towed for only a short period (e.g., less than 30 minutes), it may be sufficient to group all data into a single event record for the entire tow. However, if the VPR was towed for longer periods (e.g., for over 24 hours as was the case of the pilot dataset), a grouping for the entire tow may result in loss of important details. In the pilot dataset, the software itself produced a grouping every 4 seconds.

**Figure 17.**
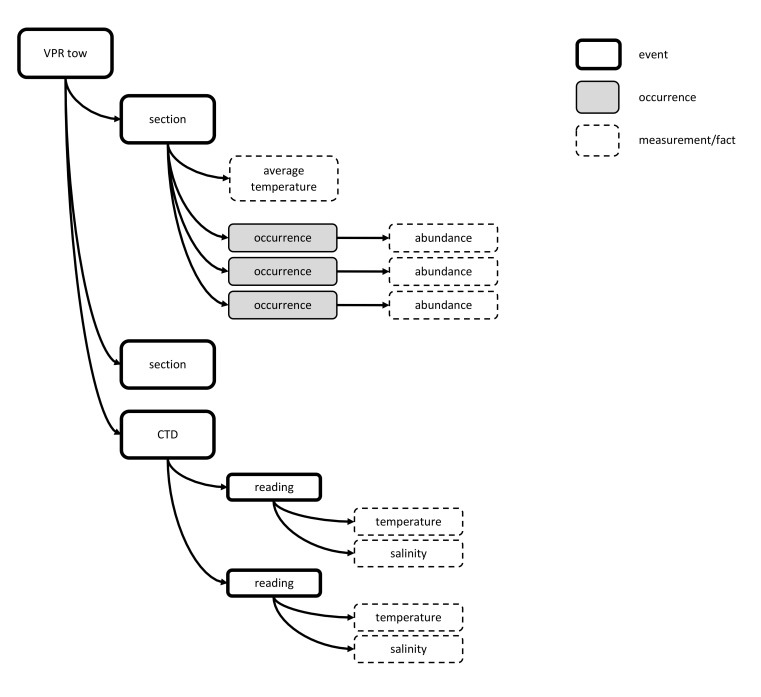
A schematic presentation showing how VPR data collected together with a CTD sensor can be stored using Option 6. In the example the bold rectangles are sampling events, the dashed rectangles measurements or facts, the grey rectangles are occurrences. Software can group the occurrences from all images taken in a defined interval into a "section" event. Derived abiotic data (like average temperature) from a sensor can be linked to this interval. Additionally - if preferred - the raw CDT data can be stored as a seperate child event "CTD" of the VPR tow (as shown in this figure).

Option 6 can capture such data when one considers each grouping created by the software as one event; the taxonomic information can be added to the Occurrence Extension and the abundances to the eMoF Extension, in the same way as described earlier for Event Type datasets. The event hierarchy can be used to group all events belonging to the same tow as is illustrated by Fig. 17.

As is true for some other types of sensors (e.g., Zooscan, Underwater Vision Profiler, etc.), the data obtained by a VPR are derived from images. Links to images can be included in the DwC-A format using the DwC Simple Multimedia Extension. The easiest way to do this for the VPR dataset would be to store all images associated to the entire tow as a .zip file in an archive. A Simple Multimedia record linking to the parent event can then provide a link to this archive.

However, it may be more useful to link each individual image to its associated occurrence record. The Occurrence Extension contains a DwC term *associatedMedia*, appropriate to include URL links to such images. However, this latter option does not seem compatible with the grouping approach worked out above.

## The custom ExtendedMeasurementOrFact Extension of option 6

The ExtendedMeasurementOrFact (eMoF) Extension is a new extension developed during the workshop. It extends the DwC MeasurementOrFact Extension with new terms occurrenceID and measurementTypeID. The eMoF is registered with GBIF at http://rs.gbif.org/extension/obis/extended_measurement_or_fact.xml. It contains the following columns:

ID: identifier used by the DwC-A standard to link the eMoF to the Core file.

measurementID: An identifier for the MeasurementOrFact (information pertaining to measurements, facts, characteristics, or assertions). May be a global unique identifier or an identifier specific to the data set. See: http://rs.tdwg.org/dwc/terms/index.htm#measurementID

occurrenceID (***new***): The identifier of the occurrence the measurement or fact refers to. If not applicable, it should be left empty. See: http://rs.tdwg.org/dwc/terms/occurrenceID

measurementType: The nature of the measurement, fact, characteristic, or assertion. Recommended best practice is to use a controlled vocabulary. See: http://rs.tdwg.org/dwc/terms/measurementType

measurementTypeID (***new***): An identifier for the measurementType (global unique identifier, URI). The identifier should reference the measurementType in a vocabulary. See: http://rs.iobis.org/obis/terms/measurementTypeID

measurementValue: The value of the measurement, fact, characteristic, or assertion. See: http://rs.tdwg.org/dwc/terms/index.htm#measurementValue

measurementValueID (***new***): An identifier for facts stored in the in the column measurementValue (global unique identifier, URI). This identifier can reference a controlled vocabulary (e.g. for sampling instrument names, methodologies, life stages) or reference a methodology paper with a DOI. When the measurementValue refers to a value and not to a fact, the measurementvalueID has no meaning and should remain empty. See: http://rs.iobis.org/obis/terms/measurementValueID

measurementAccuracy: The description of the potential error associated with the measurementValue. See: http://rs.tdwg.org/dwc/terms/index.htm#measurementAccuracy

measurementUnit: The value of the measurement, fact, characteristic, or assertion. Recommended best practice is to use the International System of Units (SI). See: http://rs.tdwg.org/dwc/terms/index.htm#measurementUnit

measurementUnitID (***new***): An identifier for the measurementUnit (global unique identifier, URI). The identifier should reference the measurementUnit in a vocabulary. See: http://rs.iobis.org/obis/terms/measurementUnitID

measurementDeterminedDate: The date on which the MeasurementOrFact was made. See: http://rs.tdwg.org/dwc/terms/index.htm#measurementDeterminedDate

measurementDeterminedBy: A list (concatenated and separated) of names of people, groups, or organizations who determined the value of the MeasurementOrFact. See: http://rs.tdwg.org/dwc/terms/index.htm#measurementDeterminedBy

measurementMethod: A description of or reference to (publication, URI) the method or protocol used to determine the measurement, fact, characteristic, or assertion. See: http://rs.tdwg.org/dwc/terms/index.htm#measurementMethod

measurementRemarks: Comments or notes accompanying the MeasurementOrFact. See: http://rs.tdwg.org/dwc/terms/index.htm#measurementRemarks

Adding the column occurrenceID allows for an additional link between the Occurrence Extension and the eMoF Extension. The eMoF Extension can be used in combination with the Event Core and the Occurrence Extension to capture both abiotic measurements, linked only to the eventID, and biotic measurements, linked both to the eventID and the occurrenceID.

The additional columns measurementTypeID, measurementValueID and measurementUnitID will help with data standardisation. These columns should contain a persistent identifier which links to more information about the measurement type, the measurement fact or the measurement unit in an externally controlled vocabulary.

The addition of measurementValueID may seem strange at first. However, one needs to remember that the eMoF Extension can also be used to store facts. Some facts such as sampling instruments, sampling platforms and life stages can also refer to a controlled vocabulary. When a measured (quantitative) value is stored, the measurementValueID has no meaning and should remain empty.

The eMoF Extension creates the opportunity to semantically store and document all types of measurements and facts related to events and occurrences. We propose here to fully take advantage of this and use the eMoF to store organism quantifications (e.g. abundance, wet weight biomass, % live cover), species biometrics (e.g. body length), facts documenting a specimen (e.g. living/dead, behaviour, trophic status), facts documenting the sampling activity (e.g. sampling device, sampled area, sampled volume, sieve mesh size) and abiotic measurements (e.g. temperature, salinity, oxygen, sediment grain size, habitat features). We discuss the more controversial ones in detail:

- Organism quantifications: Although the Occurrence Extension includes the terms organismQuantityType and organismQuantity, storing the quantification in the eMoF has several advantages. It allows capturing both individual counts and derived quantifications (abundance or biomass) without the need to duplicate occurrence records, as discussed in several previous sections. Furthermore, the eMoF Extension, through the measurementTypeID field, enables reference to external controlled vocabularies to standardize parameters, rather than maintaining a vocabulary within OBIS. The eMoF Extension removes limits on the number of parameters stored, enabling inclusion of all relevant parameters.
- Sampling activity: Characteristics such as the sampling device, the mesh size of a plankton net or a sieve and the volume of water which flowed through the sampling device are currently either not included in the DwC-A file, only documented or referenced in the EML metadata, or at best awkwardly included in the column samplingProtocol, samplingEffort or dynamicProperties. Obtaining information in this format can therefore often be complex, limited, time consuming, and prone to misinterpretation or error. Including facts such as sampling device separately in the eMoF Extension will allow standardisation of sampling instruments using the column measurementValueID. Although the Event Core does contain the columns sampleSize and sampleSizeUnit, it was felt that the eMoF Extension would be better suited to store the sampled area and/or volume because in some cases sampleSize by itself may not be detailed enough to allow interpretation of the sample. In the case of a plankton tow, the volume of water that passed through the net is relevant. In case of Niskin bottles, the volume of sieved water is more relevant than the actual volume in the bottle. In these examples, as well as generally when recording sampling effort for all protocols, eMoF enables greater flexibility to define parameters, as well as the ability to describe the entire sample and treatment protocol through multiple parameters.

The eMoF Extension was developed to be used in combination with the Event Core and Occurrence Extension, but it can also be used in combination with the Occurrence Core.

## Standardisation of measurements

The OBIS-ENV-DATA workshop identified three types of information that will be supported by the new eMoF Extension: (i) information related to sampling method and sampling effort; (ii) measurements linked to a biological occurrence and (iii) environmental measurements. Each of these will be described using a descriptive text string (measurementType); a value (measurementValue) using either a text string for facts or a numeric value for measurements; and, in the case of measurements, a unit expression (measurementUnit). These fields are completely unconstrained and can be populated with free text annotation. While free text offers the advantage of capturing complex and as yet unclassified information, the inevitable semantic heterogeneity (e.g. of spelling or wording) becomes a major challenge for effective data integration and analysis.

Standardisation of descriptive terms that capture important observations and metadata elements is being addressed through the addition of three new terms: measurementTypeID, measurementValueID and measurementUnitID. These fields will be populated using controlled vocabularies referenced using Unique Resource Identifiers (URIs). Mapping free text to a controlled vocabulary is time-consuming so it is important to select controlled vocabularies that are structurally interoperable; managed through reliable web services; benefit from good community support; and can provide access to additional mapped resources. The recommended practice is to select vocabularies that are governed according to international W3C standards (W3C 2015) in order to maximise interoperability and the access to developing tools, technologies and mapped resources including e.g. taxonomies, thesauri and multi-lingual capabilities. In addition, the use of URIs enables resolution to a more human-readable preferred label and quick access to a plain text definition.

In the environmental domain, the NERC Vocabulary Server was put in place to serve a number of domain-specific controlled vocabularies developed by the British Oceanographic Data Centre (BODC) and by the European SeaDataNet project (Leadbetter et al. 2013). The vocabularies are accessible via web services and a human-searchable interface. Three of these controlled vocabularies are of particular relevance to the OBIS-ENV-DATA project: the BODC Parameter Usage Vocabulary (P01) for annotating marine environmental and biological measurements, the BODC data storage unit vocabulary (P06) for annotating units of measurements, and the SeaVox Device categories (L22) for defining sampling instruments and sensors. All three collections are already well populated and regularly maintained by a team of scientists dedicated to responding to vocabulary requests; L22 and P01 are already used by a large multi-national community of marine data managers and scientists.

Currently no controlled vocabulary exists for sampling methods. Sampling information can be complex and highly specific. It is an essential element of the observation and is often critical for assessing the fitness for purpose of an observation. Controlled vocabularies defining concepts related to sampling methods and equipment are being developed as self-standing resources (e.g. Wiebe et al. 2014 for net deployment methods) or as part of ontologies (e.g. Diviacco et al. 2014) and these should be used whenever possible. However, concepts that describe the characteristic elements (i.e. the attributes) of a sampling method or a sampling gear needed to be defined. A new vocabulary collection identified as "OBIS sampling instruments and methods attributes" (Q01) was set up for this purpose. It is managed and served through the NERC Vocabulary Server (NVS2.0). Individual URIs associated with specific sampling characteristics can thus be used to populate measurementTypeID whenever an attribute of the sampling method is being reported.

An example on how these vocabularies can be integrated into the eMoF Extension is provided by Table 3. A selection of some terms included in the Q01 OBIS sampling instruments and methods attributes vocabulary is provided in Table 4.

## Discussion

The recommended practice proposed in this paper (option 6) has been designed specifically with marine data in mind. However, we envision that it will also prove to be beneficial for other environmental monitoring datasets facing similar problems with converting sample data to a DwC-A format. In fact, the event hierarchy - which is an important feature for OBIS-ENV-DATA - was proposed by the EU-BON community to be able to deal with complicated sampling activities like Whittaker plot design sampling (GBIF/EU BON 2015) and is already implemented by GBIF (Global Biodiversity Information Facility 2016). However, we deviate from the EU-BON approach by creating the ExtendedMeasurementorFact (eMoF) Extension in order to be able to capture all sampling characteristics as well as all biological and environmental quantifications. We feel that our approach - although more complex compared to a single file in a flat structure - is flexible and can serve multiple data types. In addition, it overcomes a current limitation (star schema) of the DwC-A format. The OBIS-ENV-DATA approach stores all parameters, which are not defined by DwC terms, in the eMoF Extension where they are more accessible than they would be if they were stored in the dynamicProperties field using a key:value encoding schema such as JSON (Wieczorek et al. 2015). The newly added occurrenceID allows the creation of a link between a measurement and the DwC Occurrence Extension. The newly added measurementTypeID, measurementValueID and measurementUnitID fields enable data standardization, and will facilitate easier data flows between OBIS and other data systems such as SeaDataNet or institutes which use ID-based vocabularies to standardize their parameters. Furthermore, the new approach, going beyond just species occurrences, will make OBIS more suited as a data sharing platform for biodiversity monitoring programmes (e.g. as part of the Global Ocean Observing System - GOOS or any upcoming Marine Biodiversity Observation Networks (MBONs) as part of the Group on Earth Observations (GEO)). Serving combined datasets will definitely be appreciated by a large user community and should enable a wider range of scientific applications.

### Future perspectives

It is possible to open up the eMoF extension by replacing the occurrenceID with a resourceID, which allows more flexiblility in linking to other extensions. This resourceID can refer to the primary ID used in any extension while the occurrenceID can only refer to the Occurrence Extension. This resourceID might even refer to IDs used in the DwC-A resource without a dedicated extension like a locationID, an organismID or a taxonID. However we did not pursue this idea further as it will increase the complexity of the DwC-A file and at this stage, the addition of a simple link to the Occurrence extension was sufficient for the problems encountered by the OBIS-ENV-DATA pilot project.

Environmental measurements are often associated with quality flags if the data have been subjected to a quality control assessment. It was suggested that a measurementQuality term be added to the eMoF and that it could be populated using the IODE Primary Level Quality Flag scheme that was designed for environmental data exchange (Paris. Intergovernmental Oceanographic Commission of UNESCO 2013). However it was decided that further discussion was needed on implementing the scheme and this could be addressed during a later phase of the OBIS-ENV-DATA project.

The OBIS-ENV-DATA pilot project proposes a new standard to efficiently capture sample event data, telemetry data, CTD data and visual sensor based data. However, there are more biological data types which are of interest to OBIS (or the community wishes OBIS to consider) and it needs to be seen whether OBIS-ENV-DATA can implement those as well. These include but are not limited to (i) length frequency data combined with abundances, (ii) data derived from stomach analyses combined with the organism in which they were found, (iii) OTU information obtained by genetic analyses of samples, (iv) biological responses to ocean acidification and (v) (passive and active) acoustic receiver data.

During the course of the OBIS-ENV-DATA pilot project several questions were raised on whether OBIS should store abiotic data together with biological data in the same DwC-A file, instead of just linking to other data systems which may be more suited to hold these types of data. The primary objective of the OBIS-ENV-DATA pilot project was to keep together the abiotic and biotic data which were (i) sampled together with the purpose to be analyzed together. These abiotic data are (ii) typically collected by a biologist, (iii) considered to be part of the biological dataset, (iv) lose a lot of their value when considered separately, (v) are rarely finding their way to a specialized environmental data system and thus (vi) are at risk of being lost. The proposed standard can handle entire CTD casts alongside biological data in a DwC-A file. However, it might be opted to only store derived CTD data such as the thermocline depth or the thickness of the thermocline layer, if the latter is preferred. In any case, it is the aim that the abiotic data captured by OBIS will also find its way to specialized regional and global repositories (e.g. SeaDataNet, World Ocean Database etc).

Exploring how to link species occurrence data in OBIS to environmental data systems falls beyond the scope of the current OBIS-ENV-DATA pilot project. However, this has of course great relevance to the project as it provides an alternative way to linking biological data with abiotic data stored in different systems. This can, for example, be done by registering sampling events as a persistent resource for linking data, which is an area of active research and development in the oceanographic science community. Many projects touch on the theme of persistent identifiers and linking between data, including the Rolling Deck to Repository project (Arko et al. 2013), the THOR project, and the Research Data Alliance. Employing such linked data methods on the ultimate parent event in the hierarchy (e.g. a cruise) or on specific sampling events can create meaningful linkages across data systems and allow for data provenance and traceability.

## Conclusion

The proposed OBIS-ENV-DATA standard is the outcome of a workshop held in October 2015 at the UNESCO-IOC project office for IODE in Ostend, Belgium. Since then, the OBIS-ENV-DATA consortium has been presenting the outcome at a number of conferences and working groups to solicit community feedback (a.o. AGU Ocean Science Meeting in New Orleans February 2016, GOOS and OBIS Steering Group meetings May 2016, IMDIS October 2016). On all occasions, the proposed standard was welcomed and very appreciated. The eMoF Extension as presented in this paper has already been brought into production by GBIF, which means it is available from registered IPTs. In addition, OBIS has adapted its harvesting procedures to be able to cope with the proposed data standard.

Before the end of the OBIS-ENV-DATA pilot project, i.e. March 2017, three crucial steps are still to be undertaken: (i) The usefulness of the proposed standard will be verified during a science workshop where invited scientists will analyse data formatted according to the OBIS-ENV-DATA standard; (ii) All technical aspects and best practices of the standard have to be defined in detail and made available as guidelines in the online OBIS manual, which will then be submitted to the IODE Ocean Data Standards and Best Practices project for adoption by IODE. This manual is the basis for training the OBIS nodes and is available to anyone wishing to contribute or use data from OBIS; (iii) In March 2017, the outcomes of this pilot project will be presented to the IOC Committee on IODE, which will decide on a second phase of the project, i.e. the further expansion and implementation aspects of OBIS-ENV-DATA.

During its second phase, the project will aim to (i) get formal adoption of OBIS-ENV-DATA by IODE, (ii) train OBIS nodes and IODE data centres, (iii) establish a data flow to specialized regional and global repositories for abiotic data captured by OBIS, (iv) the OBIS technical infrastructure will continue to be adapted to the standard, (v) it will be investigated how the standard can be further optimized and used to store new types of data of interest to OBIS and (vi) the OBIS-ENV-DATA project will continue its outreach activities and seek cooperation with GBIF and the wider scientific community.

The proposed OBIS-ENV-DATA standard will allow OBIS to move beyond species occurrence data, and start dealing with ecosystem data, or, as Philip Goldstein, chair of the OBIS data content enhancement Task Team phrased it during the 5^th^ OBIS Steering Group meeting (2016): "it will bring OBIS from the Biogeographozoic Era to the Neoecodatacene".

## Figures and Tables

**Table 1.** Overview the 13 pilot datasets submitted by the workshop participants. All datasets were converted to the OBIS-ENV-DATA format described in this paper and available from the OBIS-ENV-DATA IPT at http://ipt.iobis.org/obis-env/.

**Institute**	**Dataset Title**	**Functional group**	**Sampling gear**	**Data type**	**Biological data**	**Abiotic readings**
AntOBIS	Fish lengths and distribution during the BROKE-West survey (Van de Putte et al. 2010, Rosenberg and Gorton 2006)	Fish	RMT-1 net	CTD-like	Biometrics	Sensor based, environmental
AntOBIS	IMOS - AATAMS Facility - Satellite Relay Tagging Program - Near real-time CTD profile data (Roquet et al. 2014, Roquet et al. 2013)	Marine Mammals	CTD - GPS tracker	Telemetry	Biometrics	Sensor based
BCO-DMO	BCO-DMO: Georges Bank VPR data (Ashjian 2012)	Plankton	VPR	VPR	Abundance	Sensor based
BCO-DMO	BCO-DMO: Zooplankton abundance & biomass from MOCNESS Gulf of Maine (Wiebe 2009)	Zooplankton	MOCNESS net	Sample based	Abundance, biomass	Sensor based
CENPAT-CONICET	Ascent phase of the dives of one elephant seal (Lewis 2016)	Marine Mammals	CTD - GPS tracker	Telemetry	Presence	Sensor based
EurOBIS	North Sea Benthos Survey (Craeymeersh et al. 1986)	Marcobenthos	Grab	Sample based	Abundance, biomass	Sediment sample
EurOBIS	Hyperbenthic communities of the North Sea (Dewicke, A and Marine Biology Section (MARBIOL) – Ugent, Belgium 2014)	Hyperbenthos	Sorbe sledge, multicorer, CTD, secchi-disk	Sample based	Abundance, biomass	Sediment sample/ environmental
EurOBIS	Bird tracking - GPS tracking of Lesser Black-backed Gulls and Herring Gulls breeding at the southern North Sea coast (Stienen et al. 2014, Stienen et al. 2016)	Marine Birds	GPS tracker	Telemetry	Presence	Sensor based
MedOBIS	Benthic communities in Amvrakikos Wetlands: Mazoma, Tsopeli,Tsoukalio, Rodia and Logarou lagoons (September 2010 – July 2011) (Hellenic Center for Marine Research 2016)	Zoobenthos	Grab	Sample based	Abundance	Sediment sample/ water Sample
KMFRI	Siganids Southcoast Kenya (Wambiji 2015)	Fish	Not documented	Sample based	Biometrics	/
OBIS-USA	USGS South Florida Fish and Invertebrate Assessment Network Harvest (Robblee and Browder 2015)	Varia	Throw-trap	Sample based	Biomass	Environmental
OGS	Phytoplankton and abiotical data North Adriatic Gulf of Trieste LTER time-series (Cabrini et al. 2014, Celio et al. 2015, Comici et al. 2015)	Phytoplankton	Niskin bottle, CTD	Sample based	Abundance, biomass	Water sample
SWP-OBIS	Sizing Ocean Giants (McClain et al. 2015)	Megafauna	Not documented	Sample based	Biometrics	/

**Table 2.** An overview of the 6 options presented in this paper. The table includes an assessment against two evaluation criteria identified during the workshop namely: (i) the ease of training data managers to adopt this format and the difficulty for the secretariat (iOBIS) to implement it and (ii) whether the format meets the requirements to develop scientific applications. The technical evaluation criterion on IPT and DwC-A behaviour is assessed in the advantages and disadvantages columns.

OPTION	Description	Advantages	Disadvantages	Ease of training and implementation	Meets requirements for applications
**1**	Occurrence Core combined with MoF Extension.	Most simple solution.	Duplication of all abiotic measurements. It is not possible to store abiotic data from a sample without biotic data. If the abiotic sample was sampled at a different depth, coordinates or time, this cannot be stored. Event-hierarchy is not an option.	Easy.	Not able to capture all sample based data.
**2**	Event Core combined with Occurrence Extension and MoF Extension.	Still relatively simple. Abiotic data does not need to be duplicated. It is possible to differentiate between biotic and abiotic samples.	Problems to store biometric data and derived abundance or biomass. Occurrence records will be duplicated if a dataset has both abundance and biomass data.	Easy.	Not able to capture all biotic data.
**3**	Event Core combined with MoF Extension & Occurrence Core combined with MoF Extension.	Still relatively simple. The abiotic data does not need to be duplicated. One abiotic resource can be linked to many biotic DwC-A files.	At the time, it was not possible to create a resource with an event core without selecting an occurrence Extension using IPT. This option may be prone to errors as different parts of the same dataset are split up in different resources. Abiotic data are still separated from biotic data, which the OBIS-ENV-DATA project aims to avoid. It may pose problems for OBIS to combine the DwC-A files.	Difficulties are anticipated for OBIS to combine two different DwC-A files.	Yes.
**4**	Occurrence Core combined with MoF Extension; each event is documented as a separate record in the Occurrence Core.	The MoF Extension can be used for both abiotic and biotic measurements without data duplication.	It is rather complex. It’s conceptually awkward as occurrence core is meant to register occurrences not events. This option may pose problems for automated harvesters which do not expect this format.	Rather complex.	Yes.
**5**	Event Core combined with Occurrence Extension and MoF Extension; each occurrence is documented as a “dummy” event in the Event Core.	The MoF Extension can be used for both abiotic and biotic measurements without data duplication.	Very complex. It’s conceptually awkward as event core is meant to register sample events not occurrences.	Rather complex.	Yes.
**6**	Event Core combined with Occurrence Extension and a newly proposed MoF Extension; both Extensions are linked, based on an OccurrenceID.	Most intuitive sollution which works to capture all datatypes. The MoF Extension can be used for both abiotic and biotic measurements without data duplication. Additional fields are included in the eMoF Extension to allow for standardisation of parameters.	A new extension needs to be registered at GBIF.	Rather easy.	Yes.

**Table 3.** Examples of sampling information records in the eMoF Extension using Q01, P06 and L22.

**measurement Type**	**measurementTypeID**	**measurement Value**	**measurementValueID**	**measurement Unit**	**measurementUnitID**
Net diameter	http://vocab.nerc.ac.uk/collection/Q01/current/Q0100012/	50		cm	http://vocab.nerc.ac.uk/collection/P06/current/ULCM/
Net mesh size	http://vocab.nerc.ac.uk/collection/Q01/current/Q0100015/	200		um	http://vocab.nerc.ac.uk/collection/P06/current/UMIC/
Sampling gear name	http://vocab.nerc.ac.uk/collection/Q01/current/Q0100002/	WP-2	http://vocab.nerc.ac.uk/collection/L22/current/NETT0168/		

**Table 4.** A few examples of terms in the new (Q01) vocabulary collection "OBIS sampling instruments and methods attributes".

**Preferred label**	**Identifier**	**Definition**	**Recommended usage in eMoF Extension**
Sampling instrument name	Q0100002	The name of the gear or instrument used to collect the sample or make the in situ measurement or observation.	corresponding measurementValue should preferably point to a repository like L22; measurementUnit is left empty
Sampling device aperture width	Q0100013	The smaller dimension of the sampling area of a device with a rectangular aperture (e.g. a grab or a trawl); the type of device is specified elsewhere.	measurementValue set to width value and measurementUnit set to appropriate unit preferably from a controlled vocabulary like P06
Sampling device aperture length	Q0100014	The larger dimension of the sampling area of a device with a rectangular aperture (e.g. a grab); the type of device is specified elsewhere.	measurementValue set to length value and measurementUnit set to appropriate unit from a controlled vocabulary like P06
Sampling device aperture diameter	Q0100012	The diameter of the sampling area of a device with a circular aperture (e.g. a corer or a net); the type of device is specified elsewhere.	measurementValue set to diameter value and measurementUnit set to appropriate unit from a controlled vocabulary like P06
Sampling net mesh size	Q0100015	The mesh size of the sampling net used to obtain the sample. The type of net is specified elsewhere.	measurementValue set to mesh size value and measurementUnit set to appropriate unit from a controlled vocabulary like P06

## References

[B3386225] Arko Robert, Chandler Cynthia, Stocks Karen, Smith Shawn, Clark Paul, Shepherd Adam, Moore Carla, Beaulieu Stace Rolling Deck to Repository (R2R): Collaborative Development of Linked Data for Oceanographic Research. EGU General Assembly 2013, held 7-12 April, 2013 in Vienna, Austria id. EGU2013-9564. http://adsabs.harvard.edu/abs/2013EGUGA..15.9564A.

[B3439974] Ashjian C. (2012). Video Plankton Recorder data (formatted with taxa displayed in single column); from R/V Columbus Iselin and R/V Endeavor cruises CI9407, EN259, EN262 in the Gulf of Maine and Georges Bank from 1994-1995.. http://www.bco-dmo.org/dataset/3685.

[B3386097] Ashjian Carin J, Davis Cabell S, Gallager Scott M, Alatalo Philip (2001). Distribution of plankton, particles, and hydrographic features across Georges Bank described using the Video Plankton Recorder. Deep Sea Research Part II: Topical Studies in Oceanography.

[B3428566] Cabrini M, Fornasaro D, Virgilio D, Fonda-Umani S (2014). Phytoplankton and abiotical data North Adriatic-Gulf of Trieste LTER time-series.

[B3440067] Celio M., Comici C., Falconi C., Tamberlich F., De Vittor C., Predonzani S., Fonda Umani Serena (2015). C1-LTER-1998-2005-Physical and biogeochemical data; time-series_Gulf of Trieste; NorthAdriatic. National Institute of Oceanography and Experimental Geophysics (OGS).

[B3440121] Comici C., De Vittor C., Falconi C., Lipizer M. (2015). C1-LTER-2005-2010-Physical and biogeochemical data; time-series_Gulf of Trieste; NorthAdriatic.

[B3428526] Craeymeersh J, Kingston P., Rachor E., Duineveld G., Heip Carlo, Vanden Berghe Edward (1986). North Sea Benthos Survey.

[B3382599] Decker Cynthia J, O’Dor Ron (2002). A Census of marine life: unknowable or just unknown?. Oceanologica Acta.

[B3428596] Dewicke A, Marine Biology Section (MARBIOL) – Ugent Belgium (2014). Hyperbenthic communities of the North Sea.

[B3382758] Diviacco Paolo, Cauwer Karien De, Leadbetter Adam, Sorribas Jordi, Stojanov Yvan, Busato Alessandro, Cova Andrea (2014). Bridging semantically different paradigms in the field of marine acquisition event logging. Earth Science Informatics.

[B3385195] GBIF Darwin Core Archives – How-to Guide, version 1. http://links.gbif.org/gbif_dwca_how_to_guide_v1.

[B3385234] BON GBIF/EU Publishing sample data using the GBIF IPT. Version 23/3/2015. http://www.gbif.org/sites/default/files/gbif_IPT-sample-data-primer_en.pdf.

[B3386424] Facility Global Biodiversity Information Sample Event Data. https://github.com/gbif/ipt/wiki/sampleEventData.

[B3382589] Grassle Frederick (2000). The Ocean Biogeographic Information System (OBIS): An On-line, Worldwide Atlas for Accessing, Modeling and Mapping Marine Biological Data in a Multidimensional Geographic Context. Oceanography.

[B3382579] Grassle J. Frederick, Stocks Karen (1999). A Global Ocean Biogeographic Information System (OBIS) for the Census of Marine Life. Oceanography.

[B3428539] Research Hellenic Center for Marine (2016). Benthic communities in Amvrakikos Wetlands: Mazoma, Tsopeli,Tsoukalio, Rodia and Logarou lagoons (September 2010 – July 2011). http://doi.org/10.15468/dffi6y.

[B3386137] Leadbetter Adam M., Lowry Roy K., Clements D. Oliver (2013). Putting meaning into NETMAR – the open service network for marine environmental data. International Journal of Digital Earth.

[B3428517] Lewis M N (2016). Ascent phase of the dives of one elephant seal (OBIS-ENV example).. http://ipt.iobis.org/obis-env/resource?r=ctdascentfase.

[B3428575] McClain CR, Balk MA, Benfield MC, Branch TA, Chen C, Cosgrove J, Dove ADM, Gaskins LC, Helm R, Hochberg FG, Lee FB, Marshall A, McMurray SE, Schanche C, Stone SN, Thaler AD (2015). Data from: Sizing ocean giants: patterns of intraspecific size variation in marine megafauna. http://nzobisipt.elasticbeanstalk.com/resource.do?r=sizinggiants.

[B3385054] Nations Oceans and the Law of the Sea in the General Assembly of the United A/RES/69/245: Resolution adopted by the General Assembly on 29 December 2014. http://daccess-ods.un.org/access.nsf/Get?Open&DS=A/RES/69/245&Lang=E.

[B3385063] Nations Oceans and the Law of the Sea in the General Assembly of the United A/RES/70/235: Resolution adopted by the General Assembly on 23 December 2015. http://daccess-ods.un.org/access.nsf/Get?Open&DS=A/RES/70/235&Lang=E.

[B3386197] UNESCO Paris. Intergovernmental Oceanographic Commission of Ocean data standards. Volume 3. Recommendation for a quality flag scheme for the exchange of oceanographic and marine meteorological data (IOC Manuals and Guides, 54, Vol. 3.) 12 pp. (English.)(IOC/2013/MG/54-3). http://www.nodc.noaa.gov/oceanacidification/support/MG54_3.pdf.

[B3428557] Robblee Michael, Browder Joan A. (2015). South Florida Seagrass Fish and Invertebrate Assessment Network (FIAN)- Harvest. https://www.sciencebase.gov/catalog/item/53a1cc5fe4b0403a441545a5.

[B3381553] Robertson Tim, Döring Markus, Guralnick Robert, Bloom David, Wieczorek John, Braak Kyle, Otegui Javier, Russell Laura, Desmet Peter (2014). The GBIF Integrated Publishing Toolkit: Facilitating the Efficient Publishing of Biodiversity Data on the Internet. PLoS ONE.

[B3439914] Roquet Fabien, Williams Guy, Hindell Mark A., Harcourt Rob, McMahon Clive, Guinet Christophe, Charrassin Jean-Benoit, Reverdin Gilles, Boehme Lars, Lovell Phil, Fedak Mike (2014). A Southern Indian Ocean database of hydrographic profiles obtained with instrumented elephant seals. Scientific Data.

[B3439879] Roquet Fabien, Wunsch Carl, Forget Gael, Heimbach Patrick, Guinet Christophe, Reverdin Gilles, Charrassin Jean-Benoit, Bailleul Frederic, Costa Daniel P., Huckstadt Luis A., Goetz Kimberly T., Kovacs Kit M., Lydersen Christian, Biuw Martin, Nøst Ole A., Bornemann Horst, Ploetz Joachim, Bester Marthan N., McIntyre Trevor, Muelbert Monica C., Hindell Mark A., McMahon Clive R., Williams Guy, Harcourt Robert, Field Iain C., Chafik Leon, Nicholls Keith W., Boehme Lars, Fedak Mike A. (2013). Estimates of the Southern Ocean general circulation improved by animal-borne instruments. Geophysical Research Letters.

[B3439749] Rosenberg M., Gorton R. (2006). BROKE West Survey, Marine Science Cruise AU0603 - Oceanographic Field Measurements and Analysis.

[B3428474] Stienen EWM, Desmet P, Aelterman B, Courtens W, Feys S, Vanermen N, Verstraete H, Van de walle M, Deneudt K, Hernandez F, Houthoofdt R, Vanhoorne B, Bouten W, Buijs RJ, Kavelaars MM, Müller W, Herman D, Matheve H, Sotillo A, Lens L (2014). Bird tracking - GPS tracking of Lesser Black-backed Gulls and Herring Gulls breeding at the southern North Sea coast. http://doi.org/10.15468/02omly.

[B3386059] Stienen Eric W. M., Desmet Peter, Aelterman Bart, Courtens Wouter, Feys Simon, Vanermen Nicolas, Verstraete Hilbran, de Walle Marc Van, Deneudt Klaas, Hernandez Francisco, Houthoofdt Robin, Vanhoorne Bart, Bouten Willem, Buijs Roland-Jan, Kavelaars Marwa M., Müller Wendt, Herman David, Matheve Hans, Sotillo Alejandro, Lens Luc (2016). GPS tracking data of Lesser Black-backed Gulls and Herring Gulls breeding at the southern North Sea coast. ZooKeys.

[B3439758] Van de Putte Anton P., Jackson George D., Pakhomov Evgeny, Flores Hauke, Volckaert Filip A. M. (2010). Distribution of squid and fish in the pelagic zone of the Cosmonaut Sea and Prydz Bay region during the BROKE-West campaign. Deep Sea Research Part II: Topical Studies in Oceanography.

[B3386128] W3C Vocabularies. https://www.w3.org/standards/semanticweb/ontology.

[B3428548] Wambiji N (2015). Siganids southcoast kenya. http://ipt.iobis.org/obis-env/resource?r=siganids-southcoast-kenya.

[B3428508] Wiebe P (2009). Zooplankton abundance and biomass from MOCNESS tows from R/V Endeavor, R/V Oceanus EN307, OC332, OC334, EN330, EN331 in the Gulf of Maine and Georges Bank from 1997-1999. http://www.bco-dmo.org/dataset/3285.

[B3386147] Wiebe P. H., Allison D., Kennedy M., Moncoiffe G. (2014). A vocabulary for the configuration of net tows for collecting plankton and micronekton. Journal of Plankton Research.

[B3385165] Wieczorek John, Döring Markus, Giovanni Renato De, Robertson Tim Simple Darwin Core. http://rs.tdwg.org/dwc/terms/simple/index.htm.

[B3381539] Wieczorek John, Bloom David, Guralnick Robert, Blum Stan, Döring Markus, Giovanni Renato, Robertson Tim, Vieglais David (2012). Darwin Core: An Evolving Community-Developed Biodiversity Data Standard. PLoS ONE.

[B3381568] Wieczorek John, Bánki Olaf, Blum Stan, Deck John, Döring Markus, Dröge Gabriele, Endresen Dag, Goldstein Philip, Leary Patrick, Krishtalka Leonard, Tuama Éamonn Ó, Robbins Robert J., Robertson Tim, Yilmaz Pelin (2014). Meeting Report: GBIF hackathon-workshop on Darwin Core and sample data (22-24 May 2013). Standards in Genomic Sciences.

[B3382749] Board Worms Editorial (2016). World Register of Marine Species. Available from www.marinespecies.org. Accessed 14/07/2016.. http://www.marinespecies.org.

[B3382624] Yarincik Kristen, O'Dor Ron (2005). The Census of Marine Life: goals, scope and strategy. Scientia Marina.

